# Interesting Analyses of Articles in Foreign Journals

**Published:** 1864-09

**Authors:** 


### INTERESTING ANALYSES OF ARTICLES IN FOREIGN JOURNALS.

### *Parisian Medical Intelligence.*

I do not intend to go over the details of that sad affair which brought its author to a bloody end—I mean the trial of Couty de la Pommerais. After creating immense sensation and keeping the public mind in breathless anxiety, first as to the decision of the jury, and then as to whether the penalty which had been pronounced would be commuted or not, everything ended in the execution of the unfortunate man, which took place before an immense multitude, even more boisterous perhaps than it usually is on such occasions, and as little edified as ever by the ghastly spectacle. Of course, the whole details of the drama, from the first rumor of the murder to the bloody expiation, formed the bone and sinew of all newspaper chronicling during the time it lasted. Some papers, foreign to our profession, seized the occasion to expatiate on the duties and dangers of the medical profession; but the special medical journals here very sensibly abstained in general from all reflections and commentaries on the subject, and contented themselves with presenting the bare facts to their readers. That affair has given rise to many important questions, and amongst others it has revived the discussion as to the necessity of abolishing the punishment of death. A certain number of political papers took up the theme and argued in an affirmative sense; and it is said that a petition asking for the abolition of that penalty is in circulation amongst the people, and already bears more than a hundred thousand signatures. You are aware that the well-known M. Dupin, attorney-general at the Cour de Cassation, in a lengthy discourse which he delivered when the case of La Pommerais went before that tribunal, and which has, I believe added nothing to his reputation, attacked in the strongest terms the principle of life insurance, declaring it to be a barbarous custom, unworthy of a civilized people, and productive of the greatest evil. Such does not seem to be the general belief here; it is thought, on the contrary, that the principle of life insurance is a product of civilization, a wise and prudent measure, and a custom too deeply rooted to be overthrown by the spirited but specious argumentation of M. Dupin. The question has, however, been incidentally agitated here in the medical journals and among the profession whether it would not be more becoming the dignity of a medical practitioner to abstain from life insurance, and thus free himself *de facto* from the slightest suspicion of dishonesty and fraud. M. J. Guerin expressed that opinion in an article published in the *Gazette Medicale*. I believe that such, on the contrary, would be a very unwise feeling; and that the adoption of such a course, far from serving our dignity, would be a weakness unworthy of our profession. The very fact of entertaining such an idea is beneath our dignity. Such, I

am happy to say, has been the opinion of the generality of papers which took the pains of answering M. Guerin. It is not because a La Pommerais and a Palmer have disgraced their title and abused their position that the whole medical corps, numbered by thou sands, should renounce the rights and advantages which belong to all free and honest men.

During the last two months, of April and May, acute diseases of the respiratory organs still prevailed in the different hospitals of Paris, but since the beginning of this month they have steadily decreased. At the same time the eruptive fevers, and principally measles, made their appearance, particularly in the children's hospitals. There the cases of croup are now far rarer and more benign.

Scarcely had the last wreaths of smoke which ended those fiery debates on the origin and nature of vaccine vanished away into the air, at the Academy of Medicine, when that learned body again set to work, and on a subject equally exhaustless and far less useful. MM. Gavarret, Bouillaud, Barth, &c., are thundering in eloquent peals against the unfortunate M. Beau and his theory on the sounds of the heart. Why throw away so much eloquence, and of the best? M. Beau's theory had already fallen; it had enlisted but a few straggling followers—it cannot stand the test of any severe examination, anatomical, physiological, or pathological. Yet does he manfully keep his ground against his numerous opponents. It is impossible to show a better countenance in a worse cause. I am much afraid that after so many eloquent speeches and multiplied efforts, M. Beau's conviction will remain the same; and I am pretty sure that were it not for the *galerie*, his adversaries would have spared themselves the exertions they have made to plead a cause already judged by the public.

Professor Laugier, one of the surgeons of the Hotel Dieu, has recently made a most important communication to the Academy of Sciences. In an operation performed on the arm, and in which the median nerve had been severed, that skillful surgeon united by a suture the two ends of the nerve. Almost immediately after signs of sensibility were observed, and in a few days more the nerve had entirely recovered all its properties of sensation and motion. I need not insist on the importance of this case, which throws such a new light on physiological pathology of the nervous system. No longer than two weeks ago, in a discussion which took place at the Society of Surgery, it was affirmed by several members that the regeneration of the nervous tubes, which alone could cause the recovery of sensibility and motility, was the work of weeks and months, and could not immediately take place. Such, also, was the opinion of M. Brown-Sequard and of MM. Vulpian and Philippeaux. These two gentlemen published last year a memoir which received academical honors, and in which they gave the relation of different experiments they had made, the result of which is entirely opposed to that recently obtained by M. Laugier. The memoir of that eminent professor, read at the Academy of Sciences, has been the scientific event of the week.—PARIS, June 28, 1864.

*The Value of the Bromide of Ammonium as a Remedial Agent in Certain Diseases.*—The phisiological effects of the bromide of ammonium formed the subject of a communication, by Dr. Gibb, at the late meeting of the British Association for the Advancement of Science, at Cambridge. They were shown to be such as to demonstrate its value in a number of diseases in which the nervous system is functionally engaged, especially the ganglionic, accompanied by pains of a moderate character. The mucous membrane of the entire body is brought more or less under its control, according to the dose and the mode of its administration. In some of the milder forms of skin disease it has also been found to be very serviceable. As an absorbent in glandular and other enlargements, it is not inferior to its sister salt, the bromide of potassium, whilst it is superior in some respects in the treatment of some other forms of disease. Dr. Gibb has under his care at the present time several cases of epilepsy in which very marked benefit has ensued from its use, in arresting and diminishing the number of fits. In mild forms of oophoritis, with its attendant symptoms, the bromide of ammonium has sometimes dispelled the latter as if by magic. Trembling, nervousness, and general uneasiness, quickly subside under its use. It acts as an antispasmodic in this respect; for it not only calms irritation, but allays nervous excitability. This has been particularly observed in various experiments, and affords sufficient encouragement to proceed with a trial in more severe cases of nervous malady than have passed through Dr. Gibb's hands.

Its effects on the mucous membrane of the eye were tested in treating cases of strumous opthalmia in the young. These were found to be remarkably beneficial and decided. In one instance, that of a girl aged twelve, the subject of strumous conjunctivitis and corneitis, with leucoma and other opacities, accompanied by great intollerance of light, which had resisted treatment, off and on, for five years, a cure resulted in five weeks under small doses of this salt; and, what was hardly expected, the opacities were actually diminishing when seen seven weeks afterwards.

The bromide of ammonium possesses some absorbing influence upon atheroma, fat, and allied compounds. Individuals who took it in quantities ranging from two to ten grains, thrice a day, were either in a state of moderate corpulency, or possessed the atheromatus expression. The former, whilst the general health continued unimpaired or improved further under its influence, seemed to get thinner, their adipose development became decidedly less, the secretion from the oily sudoriferous glands was much modified and diminished, and altogether there was an improved appearance in the countenance, which the persons themselves were aware of. This was not less marked in those undergoing atheromatous changes, and there was seen in them besides a decided clearness of the eye; the face was brighter, the integument not being so greasy; the mind seemed more active, and the bodily energy was greater.

*Weight of the Viscera on the Right and Left Sides*.—The weight of the right side of an adult of average size is twenty-two ounces and three-quarters heavier than on the left; and as this is reduced by about seven and a quarter ounces by the contents of the stomach, there is a clear preponderance of fifteen ounces in favor of the right side; the centre of gravity of the body is therefore on the right side of the middle line.

*Lithotomy in Russia*.—According to the reports of the civil hospitals in Russia, for a given period, there had been 231 cases of lithotomy, of which 194 terminated in recovery, and 37 in death. During the same period there had been only five cases of lithotrity, all of which were successful. This marked disproportion of the cases in which lithotomy and lithotrity were employed is striking, and suggests the question whether lithotrity might not have been resorted to in some of the cases in which the more formidable and dangerous operation was employed.

*M. Salmon's Operation for Hæmorrhoids.*—This operation is intended to combine the advantages of excision and ligature, and to avoid the positive objections to both. It consists of a separation of the hæmorrhoidal tumour from the subjacent parts for about the lower three-fourths of its extent, leaving it attached by the remaining upper-fourth, which is then included in a ligature. The tumours are drawn down by means of a hook with four prongs, contrived for the purpose, and the division of the lower part of them is made with scissors. These structures are always supplied with vessels which descend from above, close beneath the mucous membrane, and the trunks being necessarily included when the upper part of the tumour is tied, all danger of bleeding from the divided branches is avoided. By this proceeding, the smallest possible amount of tissue is included in the ligature, and what i. included is as far as possible removed from the anus. This latter circumstance is believed to be a great advantage, for the membrane an inch within the anus possesses little of that acute sensibility which characterizes the integument close to that aperture. In Mr Salmon's operation, the subsequent pain and irritation are very much lessened, and in evidence of this it is rarely found to extend so far as to involve the urinary organs; whereas, retention of urine, according to experience, is the rule, rather than the exception, after the ordinary operation with the ligature.

*Strychnine in Prolapsus Ani.*—M. Foucher, surgeon to the Foundling Hospital, has lately made public the results he has obtained by means of subcutaneous injections of sulphate of strychnia in the prolapsus ani of children. This not uncommon affection is frequently found to be very obstinate, and to resist for a length of time the astringent injections and other such means ordinarily resorted to for its removal. M. Foucher's method is to insert the canula of a Pravuz syringe at a point one-third of an inch outside the anul aperture, and to inject ten drops of a solution composed of three grains and a half of sulphate of strychnia dissolved in six drachms of distilled water. In one case, after two injections, performed at an interval of twenty-four hours between each, the cure was permanent. In a second case, one application sufficed; in a third case, two injections wrought a cure; and in a fourth, a successful result was attained by one alone. M. Foucher's method is, he says, perfectly painless and exempt from danger, and is certainly worthy the attention of the profession.

*Nævus Treated by Puncture with the Hot Needle.*—Surgeon J. C. Wordsworth, of the London Opthalmic Hospital, reports a case of nævus treated in this manner with entire success. The case was that of a child eight months old, and the disease affected the whole of the left upper eyelid to such an extent that its motions were much impaired, and it was stated that the growth was rapidly increasing. The skin of the lid was of a dark red color, and completely involved in the nævus; the mucous lining, too, was found to be marked by large vessels, being evidently part of the nævus that penetrated through the tarsal cartilage. When the child cried, a considerable swelling became apparent. The case was considered well adapted for treatment by the hot needle, and the surgeon ventured to promise a complete recovery. A few inspirations of chloroform produced full anæsthesia—a condition most desirable for such an operation, for many reasons. A needle, on which is a bulb about one-third of an inch from its point, intended as a reservoir for heat, and set in a handle, was heated to dull redness in the flame of a spirit-lamp. The eyelid was raised from the globe by means of an ivory spoon, inserted into the palpebral sinus, so as to protect the eye as well as to form a firm basis for the lid. A considerable number of punctures was then made through the entire substance of the lid, the needle being heated as often as necessary during the operation. In this way all the large vessels of the lid were apparently closed by the heat of the needle, punctures being made over its entire extent, at distances of about one line apart. Wet lint was applied, and the case treated as a burn. Adhesive inflammation ensued, producing induration and subsequent contraction of the substance of the lid. In a month from the operation the nævus was reduced to a few scattered vessels over the lid which had escaped the influence of the cautery; and as they produced some deformity, and might probably increase in size and number if left, a few punctures were made around them. From that time the case required no farther treatment, the nævus having completely disappeared, leaving scarcely a trace of deformity.

*Vesico-Vaginal Fistula. Result of Fifty-Five Cases.* By J. Baker Brown, F. R. C. S.—In the author's mode of operation no clamps or bars are used. The knives employed are two—one for the right hand and one for the left. The needles, of various curves, forming a series of fourteen in number, are on the same principle as Startin's, but of rigid material. They are armed with wire, and thrust through the pared edges, great care being taken to avoid the mucous coat of the bladder. The two ends of the wire are simply twisted, and so fastened. The patient is afterwards laid on the side, and a male elastic catheter, with bag attached, kept in the bladder. She is kept quiet ten or fourteen days, and the wires removed. The operation is often completed in ten minutes. Of 55 cases treated, 53 were operated upon by the author, 1 by Mr. Nunn, and 1 by Mr. Harper. Of the total number of operations, 43 were followed by perfect cure, 1 was much relieved, 2 died, 5 were not cured, and 4 are still under treatment, with every prospect of cure. Of the 43 cures, in 24 this result followed the first operation, including the cases of Mr. Nunn and Mr. Harper; in 8 the cure occurred after the second operation; in 5 after three operations; and in 6 after more than three operations. Of the 2 fatal cases, 1 died eighteen days after the operation, apparently from exhaustion, the age of the patient being fifty-six; the other died seven days after from pyæmia.

In all of his cases the author attributes the lesion to protracted labor, and not to the use of instruments or deformity of the pelvis. He states that vesico-vaginal fistula would scarcely or never occur if a labor were not allowed to become protracted.

*Opium an Antidote to Strychnine.* By J. R. Winter, M. R. C. V. S.—As strychnine is so commonly used for the poisoning of animals, it frequently happens that our dogs, either from accident or design, get destroyed by this agent. A large dog picked up some strychnine, and showed the usual and unmistakable symptoms of having taken a large and destructive dose—curving of the back, rigid extension of the limbs, &c. In order to save pain, and with a view to kill in an easier way, a good dose of tincture of opium was immediately given. To my surprise and gratification the paroxysms appeared to subside. This encouraged me to give more opium; and in the whole he got about five drachms of the liquid opiate, seemed a little drowsy, was left to sleep, and found in an hour afterwards quite well.

Shortly after this another dog was heard at night knocking himself violently about amongst buckets, boxes, &c. He was secured, and being evidently suffering from the same active poison, I administered the like remedy to him. In this case there was more difficulty to get the animal to swallow the opium; but sufficient was from time to time got down his throat, and after four hours of dreadful suffering he likewise recovered.

*Post Partum Hæmorrhage—Transfusion—Successful Result.*—Having been summoned to a midwifery case, Mr. Thorne found that a fœtus had just been expelled, which was the result of about seven months' gestation, and had evidently been dead for some considerable time. In a few minutes symptoms of internal hæmorrhage came on, and firm pressure was made over the uterus in order to expel the placenta. This was done three times, and each time it was followed by a considerable gush of blood. Mr. Thorne consequently introduced his hand into the uterus, and finding the placenta nearly entirely adherent, he detached it, and having placed a firm pad over the uterus, all hæmorrhage ceased. The patient, however, was nearly pulseless, exceedingly pallid, and evidently suffering from the loss of blood, and in consequence brandy and water was administered; on account of vomiting, however, it was discontinued. She gradually got worse and worse; twice breathing ceased, but was restored by artificial respiration. An enema syringe being obtained, half a pint of hot brandy and water was thrown into the rectum, but without any effect; and then, seeing that she was evidently dying, Mr. Thorne proposed to her friends, as a last resource, the operation of transfusion. It was at once consented to, and a young woman having volunteered to give up a little blood, the operation was performed; but owing to the loss of some blood, and the young woman fainting, only about two ounces were gotten into the patient's median cephalic vein. This, however, seemed just sufficient to stimulate the heart to renewed action; for before beginning life was almost extinct, and the pulse at the radial could scarcely be detected, whereas ten minutes afterwards the pulsations of the temporal artery were very distinct. During the next six hours she had two injections of hot brandy and strong beef tea, and after this she was fed every quarter or half hour for the next forty-eight hours. Twelve hours after the operation there was considerable thirst and pain in the head, dry, brown tongue, pulse 112, rather full and sharp. All symptoms of re-action soon passed off. She was kept for ten days on strong fluid nourishment. On the eleventh day she ate a mutton chop, and since then she has steadily improved, and at the present time only complains of debility.

*Total Irideremia.*—John T——, aged twenty-two, a pallid but otherwise healthy man, for many years a sailor. The corneæ were clear, of normal size and shape; the scleroticæ white and healthy; the lenses, when reflected lights were thrown on them, showed deep-seated opacities; the globes were unsteady, always in motion; palpation healthy; no photophobia; no ptosis of either lid; under the ophthalmoscope no vestige of iris was to be seen. The opacities were chiefly capsular on the posterior part of both lenses, with slight central opacities on their anterior surfaces, not extending deeply into the structure of the lenses; the shaded edges of both lenses were clearly to be traced, leaving a narrow ring of red, illuminated fundus all around. The optic disc and fundi of both eyes appeared healthy; he could see to read at about six inches. As a sailor, he regularly took his duty of steering by compass, both by night and day. The sight was not improved by concave or convex glasses, or by the use of an artificial diaphragm. Objects were best seen in a subdued light. There was no imperfection in any of his family. The state was congenital. His sight does not get worse.—*Lancet.*

*Anastomosis.*—M. Sucquet has, by means of injections, shown that, independently of the capillary continuity of circulation, there exists in different regions of the body certain constant and determined connections, by means of transverse branches, between the small arteries and veins, which can be observed without the aid of the microscope. This mode of communication had already been noticed in the skin of the batrachians by M. Fred. Dubois, but its occurrence in the human body was unknown. M. Sucquet has also called attention to another curious anatomical fact, also new to science—namely, that in other cases a small artery, instead of breaking up into terminal branches like its neighbors, may be seen simply to make a bend upon itself and return parallel to its original course, having become a vein, and increasing in size as other veins, neighboring veins come to inosculate with it, when it constituted the principal afferent vessel of the part. The regions in which the above vascular peculiarities have been noticed are, the skin of the fingers (more especially at their tips) and of the thenar eminence of the wrist; also in that of the elbow; on the surface of the aponeurotic expansions of the extensor tendons; on the ligaments of the hand, wrist and elbow; in the skin of the toes and soles of the feet; in the skin of the knee, round the patella, where filiform arteries accompanied by veins, with which they communicate freely, abound; on the surface of the tibio-tarsal ligaments and those of the knee-joint; in the skin of the lips, nose, eyelids, eyebrows and ears; in the mucous membrane lining the nasal fossæ; and, lastly, in that on the tip of the tongue. The knowledge of the above facts must be most precious to the physiologist; and although the application and bearing can hardly at present be estimated, yet we may look upon M. Sucquet's investigations as a most important step towards a clearer insight into those mysterious laws which regulate the nutrition and repair of the tissues, and the maintenance or restoration of circulatory equilibrium.—*Académie Impériale de Médecine.*

*Blisters in Varicose Veins.*—Mr. Ure uses blisters successfully in this disease. He considers that they cure by causing contraction of the veins and the deposition of fibrine into the surrounding areolar structures. He has found his cures to be permanent, even in persons of advanced years.

*Embolism*.—In a communication on this subject before the Royal Medical and Chisurgical Society by Mr. Sibly, the author proceeded to describe the phenomena which followed the sudden obstruction of an artery. The complete arrest of circulation, the attempt at its restoration, and the reason why, in cases of the brain, spleen and kidney, this attempt is not successful. The partially restored circulation is characterized by a zone of enlarged vessels around, and by a low form of inflammation in the part affected. In consequence of this the nutrition of the part is damaged, and fatty granules accumulate among the cell structures, thus causing the bright yellow color which is seen in the so-called fibrinous deposits. As the circulation becomes more complete, the more plastic products of inflammation are formed, the yellow color fades, the bright zone of enlarged vessels slowly disappears, and at length a cicatrix is formed. The paper concluded with a review of the arguments for and against the theory which supposes the fibrinous obstructions to have been washed away from the cavities of the left side of the heart. The author believes that obstructions may be formed in the arteries, or that they may be washed away from the heart; and after describing the mode in which obstructions formed in these two modes are to be distinguished from each other, he proceeded to give the reasons for affirming that in all the cases mentioned in this paper the plugs had come from the heart, from the arteries, or from an inflamed lung. The chief arguments made use of for arriving at this conclusion were: the peculiar appearance and structure of the plugs; their analagous structure to warty growths on the valves of the heart; the situation at which plugs are usually met with; the condition of the artery at the obstructed part; the occurrence of several plugs in neighboring or distant vessels; the very frequent, or indeed almost constant association with fibrinous deposits in the spleen and kidney; and lastly, the arguments which are derived from a consideration of the symptoms of this form of brain disease.

*M. Trousseau's Fee.*—This physician was lately summoned to Naples, and agreed to go and see the patient for a fee of $8,000. When M. Trousseau reached Naples the patient was dead. The money was paid.

*Subcutaneous Injection of Morphia after Operation, before Restoration of Consciousness.*—Mr. Paget removed the left leg of a woman at its upper fourth by means of the circular operation, whilst the patient was under the influence of chloroform. The woman was the subject of paralytic varus, which had been submitted to a former operation, with the result of the restoration of the foot to its natural shape. It was, however, a useless limb, was the subject of ulceration and chillblains, and for the purposes of progression was of no value whatever. It caused her a great deal of misery and trouble, and she was anxious to get rid of it. To this Mr. Paget consented; and after removal, the limb was found, as is usual in such cases, to have undergone fatty degeneration, and the bones were in the same state.

Aftet the stump was dressed, a subcutaneous injection of a solu tion of a third of a grain of morphia was practised, with the view of inducing freedom from pain, and some refreshing sleep after a return to consciousness. The practice, as Mr. Paget remarked, has been in use for some time at the Middlesex Hospital, and has afforded much comfort and ease, especially after many of the more important and painful operations. From a quarter to a third of a grain, or, if necessary, even half a grain, of the morphia may be employed, according to circumstances.

*Opening in Septum Ventriculorum.*—Mr. James Stedman, in a case of cyanosis in a child two years old, found, at the autopsy, warty vegetations on the semilunar valves at the base of the pulmonary artery, the orifice of that vessel being very much contracted, though patent to a certain extent. The septum ventriculorum, at its upper part or base, was deficient to about a fifth of its depth, this free border or base being concave in its long diameter, and rounded and smooth in a transverse direction—that is, passing from ventricle to ventricle.

*Black Cataract.*—Another case of this rarity has turned up, or rather turned out, in the world. It was extracted by Mr. Hulme, who believed the color was due to imbibition of the coloring matter of the blood. It had been said that these lenses contained iron.

*Small-Pox and Manslaughter.*—In a recent report by Dr. Lankester on the health of the parish of St. James, that physician referred to the prevalence of small-pox in his district. The extent to which this fatal and loathsome disease prevails is, indeed, disgraceful to our civilization. By the neglect of vaccination this malady is perpetuated amongst our population: and in England, the birth-place of Jenner, more deaths annually occur from this cause than are recorded in twenty years in countries, such as Denmark, where vaccination is strictly enforced.

Dr. Lankester made one striking and important observation on this form of preventable malady. He remarked that it was a maxim of the law, that when any person, by the commission of any illegal act, or the omission of one legally incumbent upon him, was instrumental in another's death, he was liable to be charged with and punished for manslaughter. Now, vaccination is, by law, compulsory; and when, through neglect of a parent to take this precaution, a child is attacked with small-pox, it would seem to be no strained deduction to assert that such a parent is legally and morally guilty of manslaughter, and should, on public grounds, be punished for the offence.—*Lancet.*

*Hospital Changes in Paris.*—Hospital physicians must, in Paris, retire at 65, and surgeons at 60. This regulation gives rise pretty often to promotion among the staff of hospitals. The latter is elected by competition, the successful candidates forming, at the central nosocomial office, a nucleus of officers ready to act as substitutes, or take vacant places. They are besides busily engaged, several hours a day, in admitting for the different hospitals, patients who apply, as they are expected to do, at the central office. The latest changes have been caused by the resignation of M. Malgaigne, at the Charité, and the retirement, in virtue of the above-mentioned regulation, of M. Gibert, at St. Louis. M. Malgaigne's resignation has taken every one by surprise, and is universally regretted.

*Iodide of Ammonium in Scrofulous Glands.*—The iodide of ammonium is highly recommended by English authorities, for scrofulous enlargements of the absorbent glands. Dr. Price speaks of it as having succeeded in his hands when other remedies prominent in hygiene and medicine had failed. The dose internally is from one to six grains repeated thrice daily before meals. The quantity used internally is one drachm to an ounce of lard or glycerine to be rubbed, night and morning, over the swelling. Dr. Price considers it one of the best compounds of iodine, and says there are two circumstances especially to recommend it: the stomach is seldom affected by its continued use, and its external application does not stain the skin.

*Arsenious Acid in Large Doses in Fever—A Substitute for Quinine.* By J. Turner, Surgeon Bombay Artillery.—The author has employed arsenious acid for twenty years in the treatment of intermittent fevers, and on account of the great drain upon the cinchona tree, its failure in India, and his strong opinion as to the equal if not greater value of arsenious acid in the above-named diseases, he now brings the results of his experience before the profession. He considers the fears of an inconvenience or danger arising from the remedy as much exaggerated, and instances the case of a child of nine months, to whom he gave twenty minims of the arsenite of potash within ten hours, repeating the dose on the following day, with the only effect of curing an obstinate quotidian intermittent, Mr. Turner's success was so marked, that the Director General stated that Mr. Turner should be thanked for "drawing attention to his successful treatment of intermittent fevers by large doses of arsenic, and steps should be taken by circular to urge an extended trial of this remedy, and reports requested." The course usually adopted by the author was to give the arsenite of potash as in the following prescription: Arsenite of potash and compound tincture of cardamoms, of each half a drachm; gum mucilage, three drachms; camphor mixture or water, half an ounce; mix. To be given every second hour four or five times, the last to anticipate the expected paroxysm at least two hours.

*Arsenic Smoking in Asthma.*—This is a practice of the Chinese. A French lady troubled with this affection, commenced by smoking a quarter of a grain three or four times daily in a cigaretta, and this she continued to do for about fourteen days, with the greatest benefit to her breathing and general health. She has subsequently much increased the dose, and when she feels an attack of asthma coming on, she does not weigh the arsenic, but takes up what she considers a sufficient dose on a paper knife. On weighing one of these doses I found it to be a little over three grains, and analysis proved it to be pure arsenious acid. She does not inhale the fumes and blow them out as in ordinary smoking, but when her mouth is full she swallows the smoke. The only ill effects she has ever experienced is swelling of the eyelids, and, at first, slight pricking pains about the stomach, but never to any troublesome extent. Any expected attack, now at long intervals, is cut short by a resort to the remedy.

*Treatment of Diabetes.*—M. Démeaux, whose name is already well known to the profession in connection with the introduction of coal-tar into therapeutics, read a memoir before the Academy of Sciences, at a late sitting, upon a method of treating diabetes, from which he has derived most encouraging results. "I have been," says this practitioner in his communication, "for several years past in the habit of using, in cases of diabetes mellitus, a mixture of extract of rhatany and calcined alum in equal parts, and have been so far successful as to think it incumbent upon me to call the notice of the profession to the effects I have witnessed. In all cases I have obtained a decided modification and amelioration of the symptoms, and in two instances in which the disease was well marked, a continuance of the above treatment brought about an entire cure." Two cases, it is true, are not conclusive; but as M. Démeaux has pledged himself to a more complete examination of the subject, the negative or positive solution of the question will be shortly forthcoming.

